# microRNA-491-5p protects against atherosclerosis by targeting matrix metallopeptidase-9

**DOI:** 10.1515/med-2020-0047

**Published:** 2020-06-02

**Authors:** Zhonghan He, Yayun Wang, Qin He, Manhua Chen

**Affiliations:** Department of Cardiology, The Central Hospital of Wuhan, Tongji Medical College, Huazhong University of Science and Technology, Wuhan 430030, China; Department of Cardiology, The Central Hospital of Wuhan, Tongji Medical College, Huazhong University of Science and Technology, No. 26 Shengli Street, Jiang'an District, Wuhan 430030, Hubei Province, China

**Keywords:** atherosclerosis, miR-491-5p, MMP-9, hVSMCs, proliferation, apoptosis, migration

## Abstract

Abnormal proliferation and migration of vascular smooth muscle cells (VSMCs) are critical processes that are involved in atherosclerosis. The aim of this study was to explore the role of microRNA-491-5p (miR-491-5p) in the progression of atherosclerosis by regulating the growth and migration of VSMCs. In this study, we showed that the expression of miR-491-5p was downregulated in the atherosclerotic plaque tissues and plasma samples of the patients with atherosclerosis. The bioinformatic analysis and dual-luciferase reporter assay identified that matrix metallopeptidase-9 (MMP-9) was a target gene of miR-491-5p. The results showed a significant upregulation of MMP-9 in the atherosclerotic plaque tissues and plasma samples. Subsequently, the results also showed that downregulation of miR-491-5p significantly promoted the proliferation and migration of VSMCs and inhibited the apoptosis in VSMCs. Furthermore, we detected the effects of miR-491-5p mimic on the growth and migration of VSMCs, and the results illustrated that miR-491-5p mimic could inhibit the proliferation and migration of VSMCs and promote the apoptosis of VSMCs. Notably, MMP-9 plasmid could reverse all the effects of miR-491-5p mimic on VSMCs. Collectively, our study provides the first evidence that miR-491-5p inhibited the growth and migration of VSMCs by targeting MMP-9, which might provide new biomarkers and potential therapeutic targets for atherosclerosis treatment.

## 1 Introduction

Atherosclerosis is a chronic and multifactorial illness characterized by an accumulation of lipids and fibrous elements in large arteries and is responsible for most of the morbidity and mortality worldwide [1]. Vascular smooth muscle cells (VSMCs) are one of the major parts in the arterial walls [2], fulfilling multiple kinds of distinct structural and physical functions [3]. It is reported that 70% of all cells in the atherosclerotic lesions are derived from smooth muscle cells (SMCs) [4]. Recent evidence also indicates that aberrant cell functions of VSMCs such as proliferation, migration, and apoptosis are the universal pathogenesis for multiple cardiovascular diseases including atherosclerosis and restenosis after angioplasty [5]. Therefore, VSMCs plays an essential, yet understudied, role in the progression of atherosclerosis. However, the thorough molecular mechanisms underlying the role of VSMCs in atherosclerosis are far from being fully understood.

MicroRNAs (miRNAs), a class of single-stranded non-coding RNAs of 18–24 nucleotides in length, regulate thousands of mRNA targets by binding to the 3′-untranslated regions (UTR) [6]. The miRNA expression levels in circulation and in tissue are thought to be clinical biomarkers for the diagnosis and prognosis of atherosclerosis [7]. Numerous studies have demonstrated that miRNAs are involved in a variety of basic biological procedure, such as cell development, growth, proliferation, differentiation, and apoptosis [8]. Emerging evidence suggests that miRNAs play a prominent role in regulating the functions of VSMCs during atherogenesis [9]. miR-135b-5p and miR-499a-3p play a significant role in regulating the proliferation and migration of VSMCs via suppressing the expression level of myocyte enhancer factor 2C and has been considered as biomarkers in atherosclerosis [10]. A previous study has shown that hsa-miR-148b could inhibit the proliferation and migration of VSMCs by targeting heat shock protein 90 [11]. Recently, a study showed that rs1056629A/C variation led to an increased risk for atherosclerotic cerebral infarction via improving the expression of MMP-9 through influencing the binding of miR-491-5p to the polymorphic site in the 3′-UTR of MMP-9 [12]. However, the function of miR-491-5p in the cellular processes of VSMCs that are related to atherosclerosis remains largely unclear.

MMPs are a family of more than 26 different zinc (Zn^2+^)-dependent endopeptidases and are synthesized and secreted by macrophages [13,14]. MMPs are not only important in tissue remodeling [15], wound healing, and inflammation [16] but also associated with the progression of tumor and vascular disorders [17]. Recently, it was reported that MMP-9 was highly expressed in the weak regions of the atherosclerotic plaques and might be causally implicated in plaque rupture [18]. Furthermore, MMP-9 was also identified to be essential for the progression of arterial lesions through adjusting the proliferation and migration of SMCs in MMP^−^/^−^ mice [19]. Thus, these above results demonstrated that studies on MMP-9 could help provide a more penetrating comprehension of its role in the progression of atherosclerosis.

This study aimed to investigate the role and mechanism of miR-491-5p in the growth and migration of VSMCs, so as to explore its role in atherosclerosis.

## 2 Materials and methods

### 2.1 Clinical specimens collection

The atherosclerotic plaque tissues and the corresponding normal vascular tissues were collected from 30 patients with atherosclerosis (age range: 40–62 years; male/female: 15/15; total cholesterol: 6.74 ± 0.41 mmol/L; high-density lipoprotein: 1.47 ± 0.45 mmol/L; triglycerides: 1.66 ± 0.70 mmol/L; low-density lipoprotein: 4.48 ± 0.53 mmol/L) undergoing coronary artery bypass surgery. Blood specimens were also collected from 30 patients with atherosclerosis and 30 healthy individuals (age range: 38–59 years; male/female: 15/15; total cholesterol: 4.11 ± 0.40 mmol/L; high-density lipoprotein: 1.44 ± 0.33 mmol/L; triglycerides: 1.54 ± 0.48 mmol/L; low-density lipoprotein: 2.12 ± 0.42 mmol/L). All tissue and plasma samples were separated and stored in liquid nitrogen immediately until analysis. The exclusion criteria for patients with atherosclerosis in this study include asthma, chronic or acute inflammatory disease, autoimmune disease, cancer, type I diabetes mellitus, severe heart failure, and renal and hepatic dysfunction. Healthy individuals with a history of cerebrovascular accident, myocardial infarction, coronary bypass, coronary angiography with angioplasty or stenting or both, or peripheral vascular disease were excluded from the study. Informed consents were obtained from all the participants, and the protocol of this work was approved by the Ethics Committee of the Central Hospital of Wuhan, Tongji Medical College, Huazhong University of Science and Technology.

### 2.2 Cell culture and transfection

The human VSMCs were acquired from the American Type Culture Collection (ATCC, USA). The VSMCs were cultured and maintained in Dulbecco's modification of Eagle's medium (DMEM) supplemented with 10% fetal bovine serum (FBS; HyClone, USA). At the same time, 100 U/mL penicillin and 100 µg/mL streptomycin (Sangon, Shanghai, China) were mixed into the DMEM. Then, the cells were maintained in a humidified chamber with 5% CO_2_ at 37°C. The miR-491-5p inhibitor (cat no. QPG-04193), miR-491-5p mimic (cat no. QPG-04192), inhibitor control (cat. no. QPG-04191), and mimic control (cat. no. QPG-04191) were synthesized by GenePharma (Shanghai, China). Cell transfections were conducted using Lipofectamine™ 2000 transfection reagent (Invitrogen Life Technologies, CA, USA) in line with the manufacturer’s instruction. 48 h after cell transfection, cells were processed in further assays or RNA/protein extraction, respectively.

### 2.3 MiRNA target analysis and dual-luciferase reporter assay

TargetScan 7.2 (http://www.targetscan.org/vert_72/) was used to predict the potential target of miR-491-5p, and the analysis predicted that MMP-9 was a direct target gene of miR-491-5p. To investigate the results of the analysis, the dual-luciferase reporter assays were carried out. Human MMP-9 mRNA 3′UTR WT and mutated miR-491-5p human MMP-9 mRNA 3′UTR sequences were amplified by RT-PCR and then inserted into a pmirGLO vector (Promega, USA). Moreover, VSMCs were seeded into a 24-well plate and then transfected with the indicated luciferase reporter plasmid or its mutant using Lipofectamine™ 2000 (Invitrogen Life Technologies). The cells were also transfected with miR-491-5p mimic or mimic control by Lipofectamine™ 2000. After transfection for 48 h, the luciferase activity was examined using the Dual-Luciferase Reporter Assay System (Promega Corporation, Madison, WI, USA), and the results were normalized to the Renilla luciferase activity.

### 2.4 Quantitative polymerase chain reaction (qPCR)

Total RNA was isolated from VSMCs, tissue lysates, or plasma samples using TRIzol reagent (Invitrogen, Carlsbad, CA, USA). The concentrations of RNA were detected by a NanoDrop ND-1000 instrument (Thermo Fisher, MA, USA). For reverse transcription, the total RNA was reverse-transcribed into first-strand cDNA using the HiScript™ Ⅱ Q RT SuperMix (Vazyme). Then, the qRT-PCR analysis was performed with a Step one plus system (Roche Molecular Diagnostics, Pleasanton, CA, USA) using ChamQ™ Universal SYBR qPCR Master Mix (Vazyme). The reaction conditions were as follows: 35 cycles of denaturation at 95°C for 60 s, annealing at 60°C for 60 s, and chain extension at 72°C for 1 min, followed by a final extension step at 72°C for 10 min. The relative expression levels of miR-491-5p were normalized to U6, and the relative expression levels of MMP-9 were normalized to glyceraldehyde-3-phosphate dehydrogenase (GAPDH). The primer sequences for PCR are listed as follows:

miR-491-5p forward: 5′-GGAGTGGGGAACCCTTCC-3′;
reverse: 5′-GTGCAGGGTCCGAGGT-3′;
U6 forward: 5′-GCTTCGGCAGCACATATACTAAAAT-3′;
reverse: 5′-CGCTTCACGAATTTGCGTGTCAT-3′;
MMP-9 forward: 5′-AGACCTGGGCAGATTCCAAAC3′;
MMP-9 reverse: 5′-CGGCAAGTCTTCCGAGTAGT-3′;
GAPDH forward: 5′-CTTTGGTATCGTGGAAGGACTC-3′;
reverse: 5′-GTAGAGGCAGGGATGATGTTCT-3′. The relative expression levels were quantified using the 2^−ΔΔCq^ method [20].

### 2.5 Western blot analysis

VSMCs, tissue lysates, and plasma samples were treated with radioimmunoprecipitation lysis buffer (Beyotime) and centrifuged at 12,000 rpm for 30 min at 4°C to obtain the total protein. Protein concentration was assessed by the Protein Assay Kit (Beyotime, Haimen, China). Equal amounts of the proteins (25 µg) were separated by 10% sodium dodecyl sulfate–polyacrylamide gel electrophoresis and transferred onto polyvinylidene fluoride (PVDF) membranes. After transfer, the membranes were blocked with 5% non-fat milk at room temperature for 1 h and then incubated with the following primary antibodies at 4°C overnight: MMP-9 (cat. no. Ab38898; 1:1,000; Abcam) and GAPDH (as a loading control, cat. no. Ab181602; 1:1,000; Abcam). Then, the membranes were washed with phosphate buffer solution-tween-20 (PBST) four times and incubated with corresponding horseradish peroxidase–linked secondary antibody (cat. no. 7074; 1:2,000; Cell Signaling Technology, Inc.) for 1 h at room temperature. Finally, the protein bands were detected using the ECL Plus Kit (Pierce, USA), and the intensity of the bands was quantified by the Image-J software.

### 2.6 Thiazolyl blue tetrazolium bromide (MTT) assay

The MTT assay was conducted to monitor the cell viability according to the manufacturer’s protocol. First, VSMCs were seeded into 96-well plates at a density of 10,000 cells per well 24 h prior to transfection. At 48 h post-transfection, 25 μl of MTT (5 mg/mL) was added into each well and the cells were incubated for 3–4 h at 37°C. Subsequently, the supernatant was discarded, and 200 µL of dimethyl sulfoxide (KeyGen Biotech) was added to dissolve the formazan crystals. Last, the optical density was evaluated by reading the absorbance at 480 nm of each well with a VersaMax ELISA Microplate Reader (Molecular Devices, Sunnyvale, CA, USA).

### 2.7 Flow cytometry

After 48 h of incubation, the transfected cells were collected by pancreatin enzymes and centrifuged at 1,000 rpm for 5 min at room temperature. Then, the sedimented cells were resuspended and adjusted at the density of 1 × 10^6^ cells/mL. Subsequently, the cells were incubated at 37°C for 15 min in the darkness after the addition of 5 μl Annexin-V-fluorescein isothiocyanate and 5 μl propidium iodide solutions. After staining, the cells were analyzed by the flow cytometry, and the data on cell apoptosis were analyzed using the FlowJo software (version 10.0).

### 2.8 Transwell assay

The VSMCs were transfected with inhibitor control, miR-491-5p inhibitor, miR-491-5p mimic, mimic control, control plasmid, and MMP-9 plasmid. After 48 h of transfection, the VSMCs (5 × 10^4^) in serum-free medium were seeded in the upper compartment precoated with or without Matrigel (BD Biosciences). The lower compartment was added with 400 μl medium containing 10% FBS. After incubation for 16 h, the non-migrated cells were removed from the topside of the filter using a cotton bud. The migrated cells were washed with PBS and fixed with a 4% paraformaldehyde solution and then stained with 0.1% crystal violet for 30 min at room temperature, imaged, and counted. All experiments were performed in triplicate.

### 2.9 Statistical analyses

The experimental results are shown as the mean ± standard deviation (SD) and calculated using the SPSS 17.0 software. When only two groups were compared, the Student’s *t*-test was used to assess the differences between two groups. The one-way ANOVA was performed to analyze the statistically significant difference among more than two groups. *P*-values of less than 0.05 was defined as significant.

## 3 Results

### 3.1 miR-491-5p was downregulated in atherosclerotic tissues and plasma

**Figure 1:**
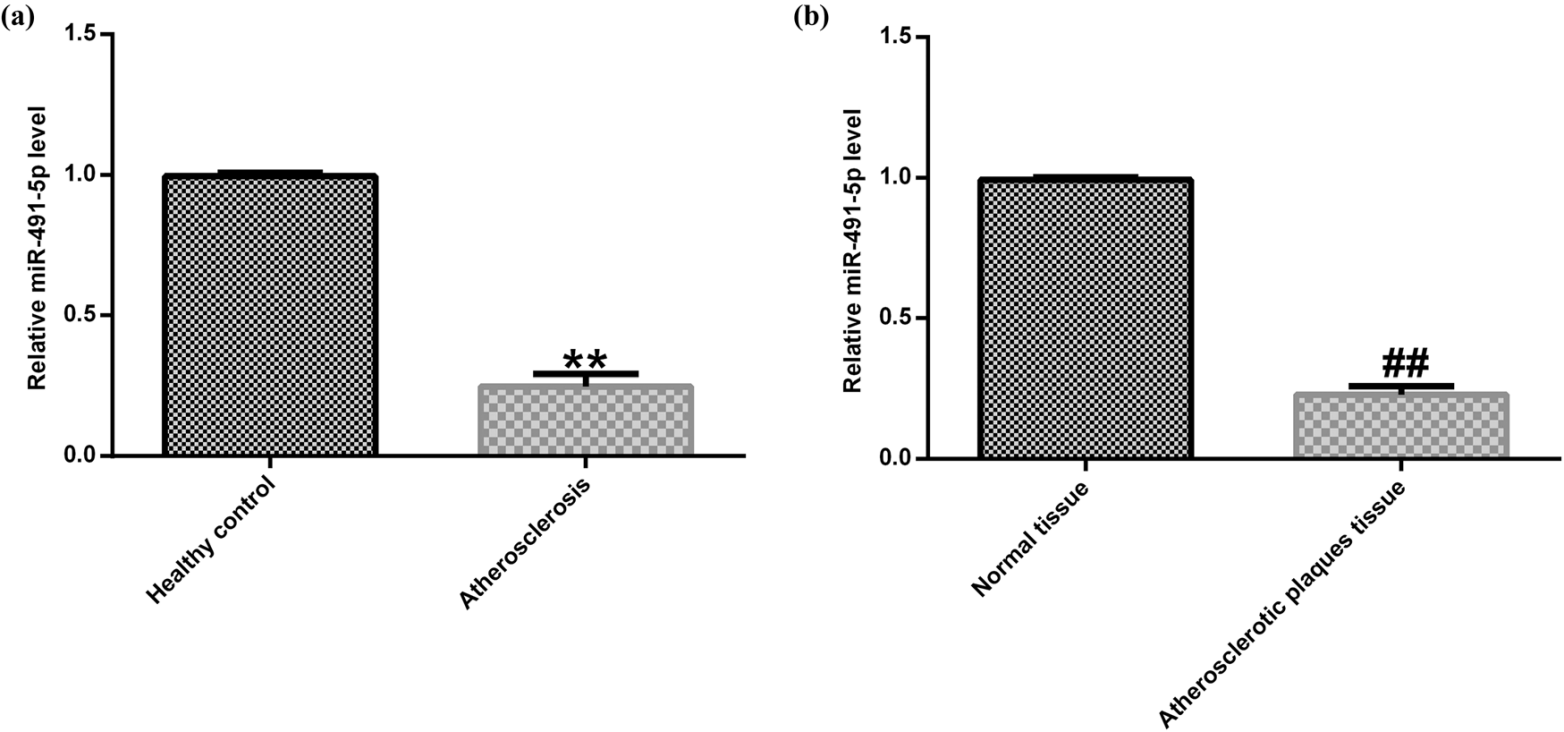
Low expression of miR-491-5p in atherosclerotic tissues and plasma. (a) Expression of miR-491-5p in the plasma samples of 30 patients with atherosclerosis and 30 controls was determined by qRT-PCR. (b) Expression of miR-491-5p in the atherosclerotic plaque tissues of 30 patients with atherosclerosis and 30 controls was detected by qRT-PCR. The data are expressed as mean ± SD; **p < 0.01 vs. healthy control; ##*p* < 0.01 vs. normal tissue.

To investigate the role of miR-491-5p in atherosclerosis development, the qRT-PCR assay was carried out to detect the miR-491-5p expression level in the atherosclerotic plaque tissues and plasma samples of the patients with atherosclerosis and in healthy individuals. As shown in Figure 1a, the expressions of miR-491-5p in the plasma samples of the patients with atherosclerosis were lower than those in healthy individuals. Similarly, the miR-491-5p expression levels in the atherosclerotic plaque tissues of the patients with atherosclerosis were lower than those in the control (Figure 1b).

### 3.2 MMP-9 was a direct target of miR-491-5p and upregulated in atherosclerotic tissues and plasma

**Figure 2:**
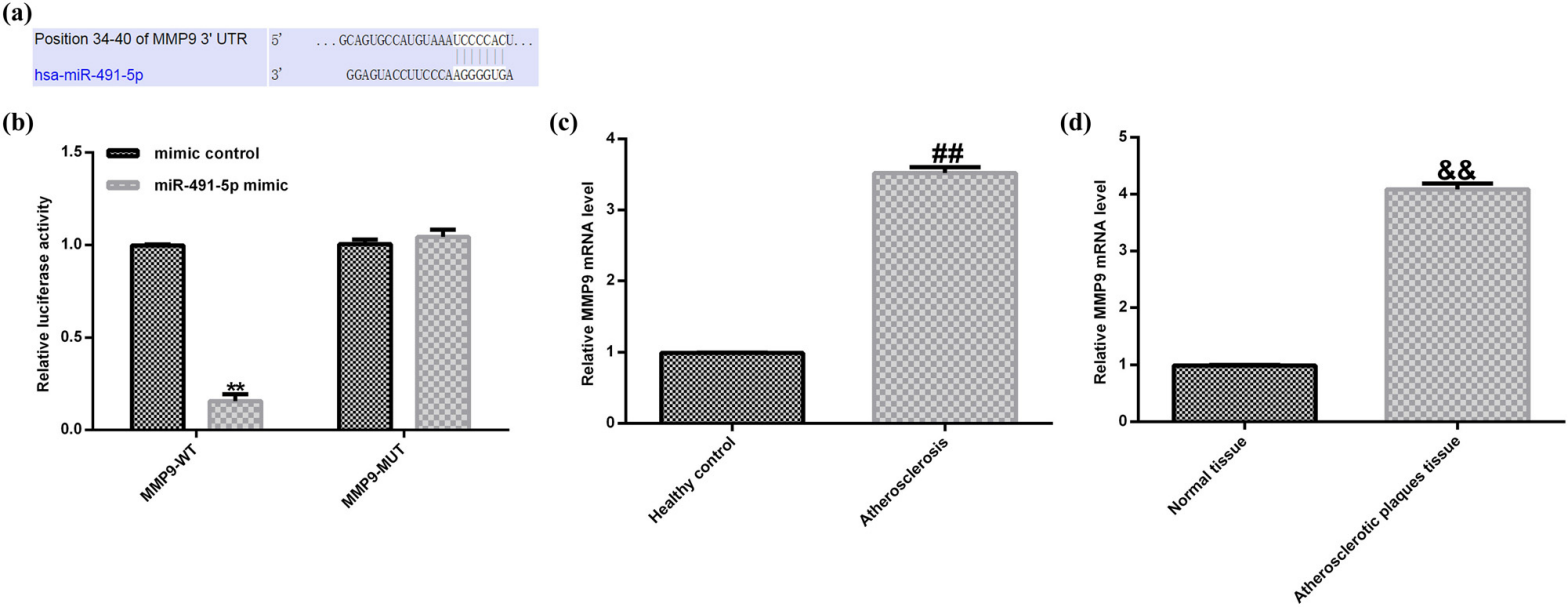
The expression of miR-491-5p that directly targets MMP-9 and MMP-9 was upregulated in the atherosclerotic tissues and plasma. (a) The predicted miR-491-5p binding site in the 3′-UTR of MMP-9. (b) The dual-luciferase analysis was used to confirm the binding sites between miR-491-5p and 3′-UTR MMP-9. (c) Expressions of MMP-9 at mRNA level in the plasma samples of the patients with atherosclerosis were detected by qRT-PCR. (d) Expressions of MMP-9 at mRNA level in atherosclerotic plaque tissues were detected by qRT-PCR. The data are expressed as mean ± SD; **p < 0.01 vs. mimic control; ##*p* < 0.01 vs. healthy control; &&*p* < 0.01 vs. normal tissue.

By using bioinformatic target gene prediction program (TargetScan), MMP-9 was predicted to be a potential target of miR-491-5p (Figure 2a). To verify the predicted binding site between miR-491-5p and MMP-9, dual-luciferase reporter constructs were used. The results demonstrated that miR-491-5p significantly reduced the luciferase activity in the wild-type 3′-UTR MMP-9 group compared with the empty vector control or the mutant 3′-UTR vector groups (Figure 2b).

To further test whether MMP-9 was involved in the miR-491-5p-mediated regulation in atherosclerosis, the qRT-PCR assay was performed. The qRT-PCR results showed that the expression level of MMP-9 was higher in the plasma samples of the patients with atherosclerosis than in the control (Figure 2c). Also, the expression level of MMP-9 was higher in the atherosclerotic plaque tissues than in the normal tissues (Figure 2d). Overall, our results strongly indicated that MMP-9 was a direct target of miR-491-5p, and it was overexpressed in the atherosclerotic tissues and plasma.

### 3.3 miR-491-5p regulated the expression of MMP-9 in hVSMCs

**Figure 3:**
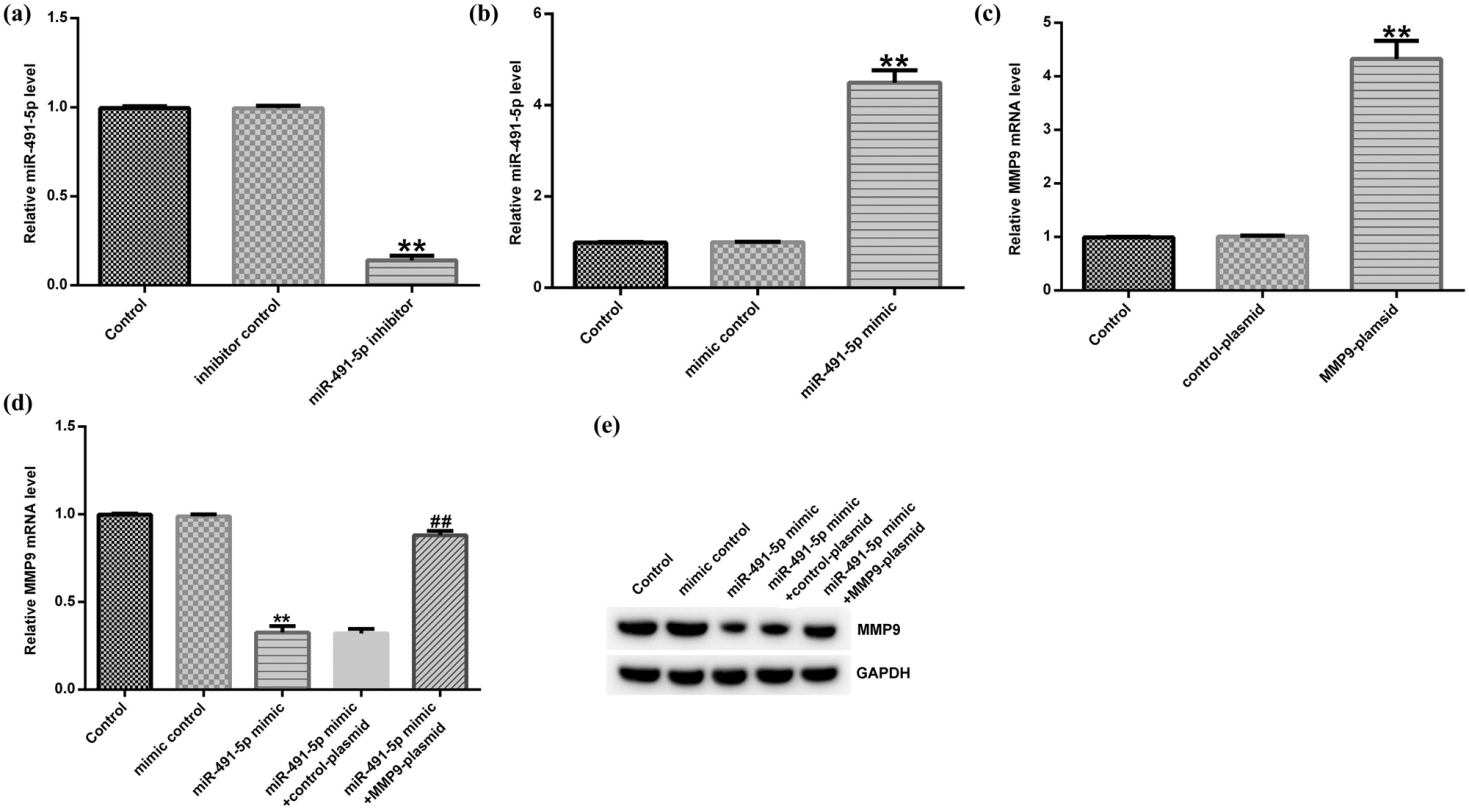
miR-491-5p negatively regulated the expression of MMP-9 in hVSMCs. (a) The level of miR-491-5p was detected by the qRT-PCR assay in hVSMCs transfected with miR-491-5p inhibitor and inhibitor control. (b) The level of miR-491-5p was detected by the qRT-PCR assay in hVSMCs transfected with miR-491-5p mimic and mimic control. (c) The mRNA level of MMP-9 was detected by the qRT-PCR assay in hVSMCs transfected with MMP-9 plasmid and control plasmid. (d and e) The mRNA and protein levels of MMP-9 were determined by the qRT-PCR and Western blot assays in hVSMCs transfected with miR-491-5p mimic accompanied with control plasmid or MMP-9 plasmid. Each data represent the mean ± SD; **p < 0.01 vs. control; ##*p* < 0.01 vs. miR-491-5p mimic.

To further investigate the relationship between miR-491-5p and MMP-9 in hVSMCs, inhibitor control, miR-491-5p inhibitor, miR-491-5p mimic, mimic control, MMP-9 plasmid, and control plasmid were transfected into hVSMCs for 48 h. The transfection efficiency was detected by the qRT-PCR analysis. As shown in Figure 3a, the expression of miR-491-5p in hVSMCs transfected with miR-491-5p inhibitor decreased remarkably compared with that in the control group. In addition, the expression of miR-491-5p was significantly higher in the miR-491-5p mimic–transfected group than in the control group (Figure 3b). Furthermore, the MMP-9 expression was increased in the MMP-9 plasmid–transfected group compared with the control group (Figure 3c). Meanwhile, we observed that the expression of MMP-9 was markedly downregulated in the miR-491-5p mimic–transfected group compared with the control group at both mRNA and protein levels, while the inhibition was reversed by MMP-9 plasmid (Figure 3d and e). The above results indicated that miR-491-5p could negatively regulate the expression of MMP-9 in hVSMCs.

### 3.4 Downregulation of miR-491-5p enhanced hVSMC viability and migration and reduced apoptosis

**Figure 4:**
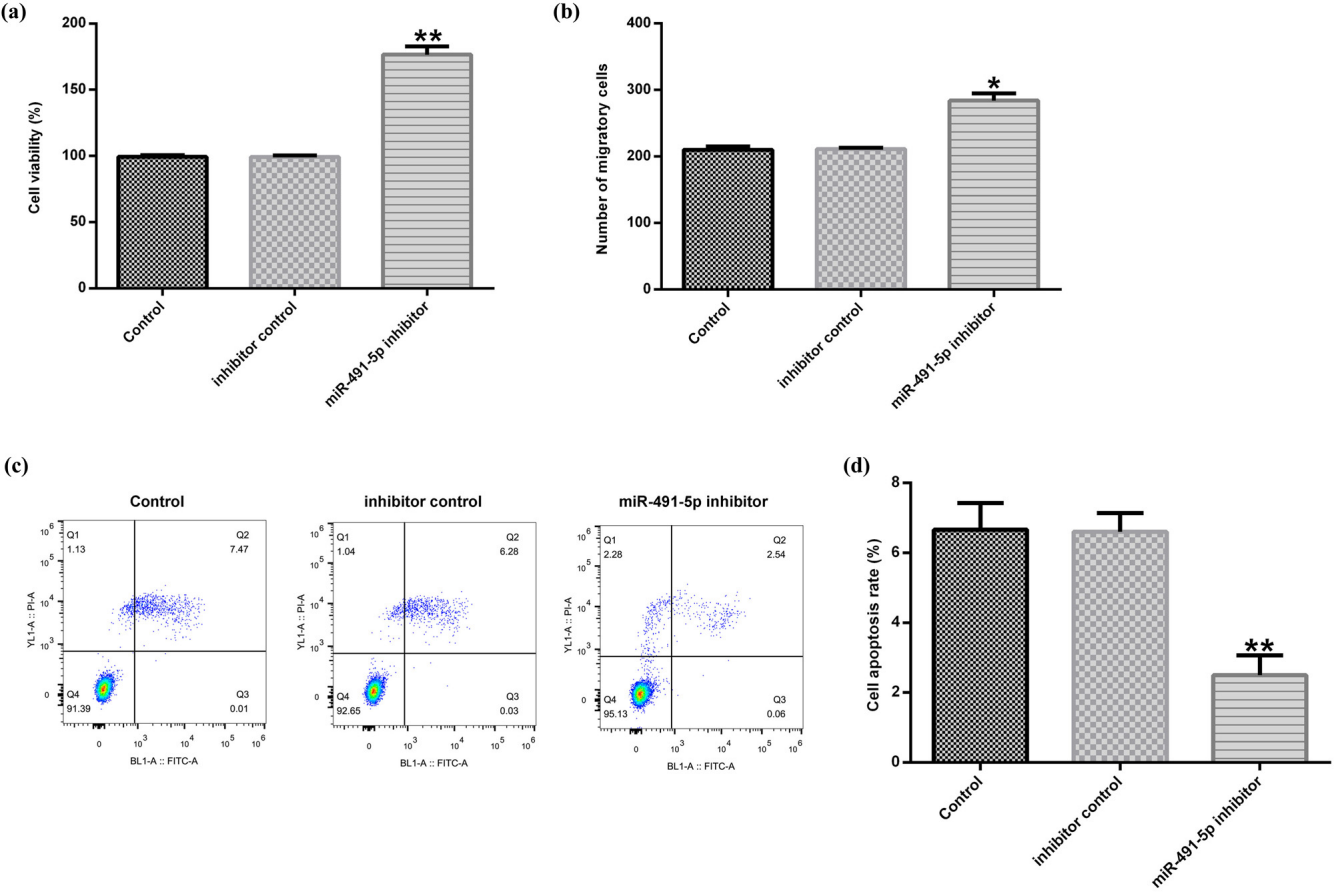
Downregulation of miR-491-5p enhanced hVSMC viability and migration and reduced the apoptosis. The hVSMCs were pretreated with inhibitor control or miR-491-5p inhibitor for 48 h. (a) The effect of miR-491-5p inhibitor on hVSMC viability was monitored by the MTT assay. (b) The effect of miR-491-5p inhibitor on hVSMC migration was determined by the transwell assay. (c and d) Apoptosis of hVSMCs was evaluated by the flow cytometric analysis. Data are shown as mean ± SD. *, **p < 0.05, 0.01 vs. control.

To further investigate the biological effects of miR-491-5p in hVSMCs, inhibitor control and miR-491-5p inhibitor were transfected into hVSMCs for 48 h. Then, the MTT assay, transwell assay, and flow cytometry analysis were used to determine the cell viability, migration, and apoptosis, respectively. The results from the MTT assay revealed that miR-491-5p inhibitor could dramatically promote the viability of hVSMCs compared with the control (Figure 4a). As shown in Figure 4b, the transwell assay demonstrated that miR-491-5p inhibitor increased the migration activity in hVSMCs. Besides, the results from the flow cytometry analysis demonstrated that the apoptosis rates of hVSMCs were decreased in the miR-491-5p inhibitor–transfected group compared with the control group (Figure 4c and d).

### 3.5 Overexpression of MMP-9 abrogated the effects of miR-491-5p on hVSMC viability, migration, and apoptosis

**Figure 5:**
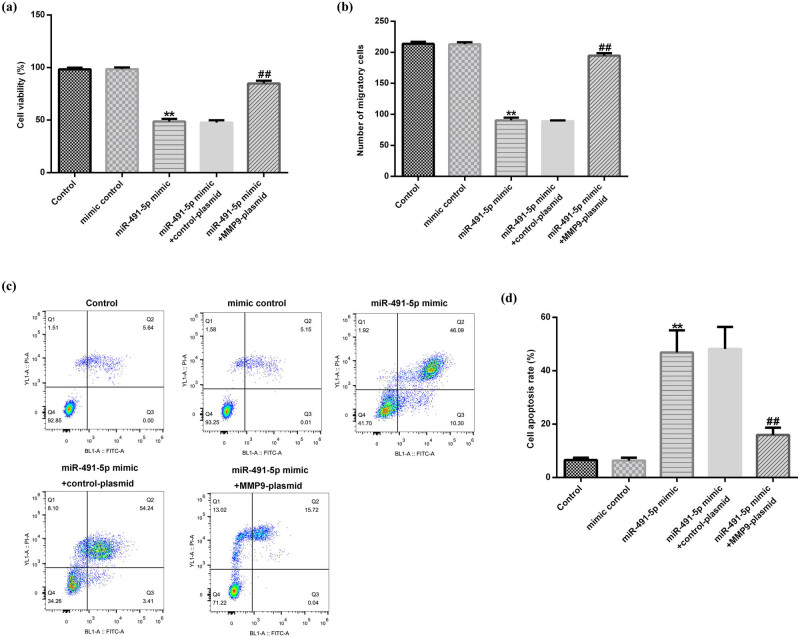
Overexpression of MMP-9 abrogated the effects of miR-491-5p on hVSMC proliferation, migration, and apoptosis. The hVSMCs were transfected with mimic control and miR-491-5p mimic alone or accompanied with control plasmid or MMP-9 plasmid for 48 h. (a) The effect of miR-491-5p mimic on hVSMC viability was monitored by the MTT assay. (b) The effect of miR-491-5p mimic on hVSMC migration was determined by the transwell assay. (c and d) The effect of miR-491-5p mimic on hVSMC apoptosis was evaluated by the flow cytometric analysis. All the effects were reversed by MMP-9 plasmid. The results are shown as mean ± SD. **p < 0.01 vs. control; ##*p* < 0.01 vs. miR-491-5p mimic.

To assess the crucial role of MMP-9 in miR-491-5p-mediated regulations of cell growth and migration in hVSMCs, experiments were carried out in hVSMCs by transfecting it with mimic control and miR-491-5p mimic alone or accompanied with control plasmid or MMP-9-plasmid. We found that miR-491-5p mimic could obviously inhibit the cell viability (Figure 5a) and migration (Figure 5b) in hVSMCs, while it promoted the apoptosis in hVSMCs (Figure 5c and d). However, all the effects were reversed by MMP-9 plasmid. Taken together, the results strongly indicated that miR-491-5p played an important role in hVSMC viability, apoptosis, and migration by targeting MMP-9 in atherosclerosis.

## 4 Discussion

Atherosclerosis is a complex disease, resulting in death in various conditions worldwide via peripheral vascular disease, myocardial infarction, and stroke [21]. Recent evidence indicated that VSMCs play an important role during the process of developing atherosclerosis [22]. The aberrant functions of VSMCs such as proliferation, migration, and apoptosis are key regulatory elements in all periods of the progression of atherosclerosis. In recent years, the emergence of miRNAs as significant regulators in the control of VSMCs proliferation and migration has attracted enhancing attention. In addition, abnormal miRNA expression profiles have been found to be involved in the process of atherosclerosis. Our study sought to identify the roles of miRNAs in the regulation of VSMCs and the identification may help improve the understanding of molecular mechanisms governing atherosclerosis and provide new diagnostic and therapeutic strategies.

In this study, we demonstrated that the expression of miR-491-5p was downregulated in atherosclerotic plaque tissues and plasma samples compared with the control. Then, the prediction program TargetScan demonstrated that MMP-9 was a direct target of miR-491-5p, and the dual-luciferase analysis further illustrated that miR-491-5p significantly reduced the luciferase activity in the wild-type 3′-UTR MMP-9 group compared with the empty vector control or the mutant 3′-UTR vector groups. MMP-9 is a member of MMPs family of at least 26 different zinc (Zn^2+^)-dependent endopeptidases and plays a necessary role in tissue remodeling, wound healing, inflammation, and vascular disorders. The involvement of MMP-9 in atherosclerotic incidents is a matter of ongoing debate, and more and more researchers have focused on the study of the effects and molecular mechanisms underlying MMP-9 in atherosclerosis. A recent study reported that MMP-9 was highly expressed in the weak regions of the atherosclerotic plaques and might be causally implicated in plaque rupture. In agreement with the previous study, our results presented that MMP-9 was highly expressed in the atherosclerotic plaque tissues and plasma samples at mRNA and protein levels.

To further investigate the relationship between miR-491-5p and MMP-9 in the progression of atherosclerosis, inhibitor control, miR-491-5p inhibitor, miR-491-5p mimic, mimic control, MMP-9 plasmid, and control plasmid were transfected into VSMCs. The results showed that miR-491-5p mimic inhibited the expressions of MMP-9 in VSMCs at both mRNA and protein levels, whereas the inhibition could be reversed by MMP-9 plasmid. In this study, we revealed that the expression of MMP-9 was regulated by miR-491-5p, which provides a possible pathway for its involvement in atherosclerosis.

Considering that the aberrant cell functions of VSMCs such as proliferation, migration, and apoptosis are associated with the development of atherosclerosis, we investigated the biological effects of miR-491-5p and MMP-9 on VSMCs. Our results demonstrated that the downregulated miR-491-5p was able to promote the proliferation and migration of VSMCs and inhibit the VSMC apoptosis, which may lead to the development of atherosclerosis. A recent study showed that upregulation of miR-362-3p inhibited the proliferation and migration of VSMCs through directly targeting a disintegrin and metalloproteinase with thrombospondin motifs 1 (ADAMTS1) [23]. Furthermore, Zhang et al. found that miR-451 inhibited the VSMC migration and intimal hyperplasia after vascular injury by the Ywhaz/p38 mitogen-activated protein kinase (MAPK) pathway [24]. Partly similar to these reports, we found that upregulated miR-362-3p could inhibit the proliferation and migration of VSMCs and promote the apoptosis of VSMCs. However, the effects were reversed by MMP-9-plasmid. These data indicated that miR-491-5p might be a vital mediator in regulating the growth and migration of VSMCs by targeting MMP-9.

In conclusion, this study provided the first evidence that miR-491-5p that targets MMP-9 is a novel modulator in the growth and migration of hVSMCs, which are related to atherosclerosis. Our findings might provide a novel insight into the molecular mechanisms underlying miRNA diagnostic and therapeutic approaches against atherosclerosis. However, there are big differences between the *in vitro* studies and atherosclerosis in humans. This study is only a preliminary *in vitro* study on the role of miR-491-5p in atherosclerosis. We had not performed *in vivo* experiments. This is a major limitation of this study, and further research is needed in the future.
